# A novel long non-coding RNA, hypoxia-inducible factor-2α promoter upstream transcript, functions as an inhibitor of osteosarcoma stem cells *in vitro*

**DOI:** 10.3892/mmr.2014.3024

**Published:** 2014-12-01

**Authors:** YONGCHENG WANG, JIE YAO, HAOYE MENG, ZHIGUO YU, ZHIGANG WANG, XUELING YUAN, HONG CHEN, AIYUAN WANG

**Affiliations:** 1 Institute of Orthopedics, Chinese PLA General Hospital, Beijing 100853, P.R. China; 2 Department of Oncology, PLA No. 161 Center Hospital, Wuhan, Hubei 430000, P.R. China; 3 Cancer Center, Division of Internal Medicine, Chinese PLA General Hospital, Beijing 100853, P.R. China; 4 Department of Oncology, Kunming General Hospital of Chendu Military Command, Kunming, Yunnan 650032, P.R. China

**Keywords:** hypoxia-inducible factor-2α, long non-coding RNA, osteosarcoma, cancer stem cell

## Abstract

Long non-coding RNAs (lncRNAs) have recently been identified as novel modulators of malignant tumors. However, the function of lncRNAs in cancer stem cells (CSCs) remains to be elucidated. The present study aimed to investigate the regulating role of a novel lncRNA, hypoxia-inducible factor-2α (HIF-2α) promoter upstream transcript (HIF2PUT), in osteosarcoma stem cells. The expression levels of HIF2PUT were assessed by quantitative polymerase chain reaction in 17 osteosarcoma tissue specimens, and the correlation between the expression of HIF2PUT and its host transcript-HIF-2α was determined. In functional experiments, HIF2PUT expression was knocked down by small interfering RNAs, or overexpressed by transfection with pcDNA-HIF2PUT, in order to evaluate the effects of HIF2PUT on cell proliferation, migration, expression rate of osteosarcoma stem cell marker CD133, and stem sphere-forming ability in MG63 cells. HIF2PUT expression levels were positively correlated with HIF-2α in osteosarcoma tissues. Overexpression of HIF2PUT markedly inhibited cell proliferation and migration, decreased the percentage of CD133 expressing cells, and impaired the osteosarcoma stem sphere-forming ability of the MG63 cells. Whereas, knockdown of HIF2PUT expression had the opposite effect. Furthermore, altering the expression of HIF2PUT resulted in a concomitant change to HIF-2α mRNA expression. These results indicate that the lncRNA HIF2PUT may be a novel regulatory factor of osteosarcoma stem cells, which may exert its function partly by controlling HIF-2α expression. Further studies regarding HIF2PUT may provide a novel therapeutic target of osteosarcoma in the future.

## Introduction

Osteosarcoma is the most common primary bone malignancy, which occurs most frequently in late childhood and early adulthood (1). In the last 25 years, the introduction of dose intensive combination chemotherapy has resulted in a five-year tumor-free survival rate of 60–75% (2). However, in patients with metastasis and local recurrence, prognosis remains poor (3). Cancer stem cells (CSCs) are a small subset of cells within malignant tumors that possess stem cell-like properties, and are responsible for cancer origination and progression (4–6). Further research on the genetic network that controls the CSC-like characteristics may provide novel strategies in cancer treatment.

Recently, a growing number of long non-coding RNAs (lncRNAs) have been identified as crucial regulators in various types of cancer (7–9). Furthermore, other lncRNAs have been shown to be associated with stem cell properties, such as pluripotency and differentiation (10). However, lncRNAs associated with CSCs have yet to be reported. Through bioinformatics analysis using public data (http://genome.ucsc.edu/), the possible cis- regulatory association between the lncRNA-mRNA pairs were predicted and the genes transcribed within a 10 kbp window upstream or downstream of lncRNAs were considered as cis target genes. If one cis target gene of an lncRNA was also a known cancer-associated mRNA, the lncRNA-mRNA pair was selected for the experiments in the present study. An lncRNA, TCONS_00004241, which is located at 2p21, on the antisense side of the promoter upstream region of a cancer-associated mRNA, hypoxia-inducible factor-2α (HIF-2α) was identified. This class of lncRNAs are known as promoter upstream transcripts (PROMPTs), which appear to regulate the transcriptional activity of host PCGs (11–14). Therefore, the lncRNA TCONS_00004241 was termed HIF-2α promoter upstream transcript (HIF2PUT). Transcript factors chip-seq data from encode/analysis (http://genome.ucsc.edu/) also demonstrated that many cancer-associated transcription factors interact with the HIF2PUT locus, particularly at the promoter region, which HIF2PUT shares with its mRNA partner, HIF-2α.

HIF-2α has previously been demonstrated to be associated with stem cell-like properties in stem cells, and in the CSCs of numerous types of cancer (15–18); therefore the present study aimed to explore the possible function of HIF2PUT in osteosarcoma CSCs, and the association with its host PCG/HIF-2α.

## Materials and methods

### Patient samples

Patients with osteosarcoma, who underwent initial surgery at the Chinese PLA General Hospital between 2012 and 2013, were retrospectively selected for the present study (Table I). Prior to resection none of the patients had received therapy. A total of 17 pairs of osteosarcoma samples and adjacent tissues were used. The use of tumor material for research was approved by the Ethical Committee of PLA General Hospital (Beijing, China). Written informed consent was obtained from the patient/the patient’s family.

### Cancer cell lines

The SAOS2, MG63, and U2OS human osteosarcoma cell lines were purchased from American Type Culture Collection (Manassas, VA, USA). The OS-732 osteosarcoma cell line was obtained from the Cell Culture Center of Peking Union Medical College (Beijing, China). The cells were cultured in Dulbecco’s modified Eagle’s medium (DMEM; Gibco Life Technologies, Carlsbad, CA, USA), supplemented with 10% fetal bovine serum (Invitrogen Life Technologies, Carlsbad, CA, USA), at 37°C in an atmosphere containing 5% CO_2_.

### Quantitative polymerase chain reaction (qPCR)

Total RNA was isolated from the osteosarcoma tumor tissues, matched adjacent normal tissues and osteosarcoma cells using TRIzol^®^ reagent (Invitrogen Life Technologies). cDNA synthesis was performed with 2 μg total RNA using the RevertAid™ H Minus First Strand cDNA Synthesis kit (Takara Bio, Inc., Otsu, Japan). The primers were obtained from Sheng Gong (Shanghai, China), and the sequences are included in Table II. A qPCR was performed using the SYBR^®^ PrimeScript RT-PCR kit (Takara Bio, Inc.) and the Applied Biosystems 7500 Fluorescent Quantitative PCR system (Applied Biosystems Life Technologies, Foster City, CA, USA). The reaction mixtures were incubated at 95°C for 30 sec, followed by 40 amplification cycles of 95°C for 5 sec and 60°C for 34 sec. The comparative cycle threshold method was used to quantify the relative expression levels of mRNA and lncRNA (19). Expression levels of the housekeeping gene β-actin were used to normalize the expression levels of the genes-of-interest. The expression levels of the genes were calculated in each patient using the following ratio: target in cancerous tissue/target in non-cancerous tissue [R(C/N)].

### Overexpression of HIF2PUT

The cDNA-HIF2PUT plasmid was constructed by introducing a *Bam*HI-*Eco*RI fragment containing HIF2PUT cDNA, into the same site in a pcDNA3.1 vector, which was obtained from GenePharma Ltd. (Shanghai, China). MG63 cells, which have low expression levels of HIF2PUT, were transfected with pcDNA-HIF2PUT using Lipofectamine^®^ 2000 (Invitrogen Life Technologies), according to the manufacturer’s instructions. The cells were collected following transfection for RNA isolation, and cell proliferation, scratch wound healing invasion and spheroid formation assays.

### Transfection of siRNA

For gene knockdown analysis, small interfering (si)RNAs targeting the lncRNA-HIF2PUT sequence, and non-targeting siRNA, were obtained from GenePharma Co., Ltd. (Shanghai, China). The siRNAs used in the present study had the following sequences: HIF2PUT siRNA1, sense 5′-CCUGCCACAUGCCUUAUCUTT-3′, and antisense 5′-AGAUAAGGCAUGUGGCAGGTT-3′; and HIF2PUT siRNA2, sense 5′-GUCUAUAUCUCUCCCUUUATT-3′, and antisense 5′-UAAAGGGAGAGAUAUAGACTT-3′. Approximately 5% MG63 cells were plated in each well of 12-well plates, at least 24 h prior to transfection, in order to achieve 30–50% confluence. siRNA transfection was performed using the X-tremeGENE Transfection reagent (Roche Diagnostics, Basel, Switzerland), according to the manufacturer’s instructions. The cells were collected following transfection for RNA isolation, and cell proliferation, scratch wound healing, invasion and spheroid formation assays.

### Flow cytometry (FCM)

The expression rate of osteosarcoma stem cell surface marker CD133 was analyzed by fluorescence-activated cell sorting (FACS) using a FACSVantage™ flow cytometer (BD Biosciences, San Jose, CA, USA). Following treatment with FcR Blocking reagent (Miltenyi Biotec, Bergisch Gladbach, Germany), the MG63 cells were incubated with phycoerythrin-conjugated anti-CD133 (Miltenyi Biotec) at 4°C for 15 min. Dead cells were eliminated using 50 μg/ml propidium iodide (EMD Millipore, Billerica, MA, USA). The labeled cells were then analyzed and separated by FCM, and the data were analyzed using CellQuest™ software (BD Biosciences). Gating was implemented based on negative-control staining profiles.

### Cell proliferation assay

Following transfection, cell proliferation was assessed using an MTS assay (Promega Corporation, Madison, WI, USA), according to the manufacturer’s instructions. MG63 cells (2×10^3^ cells/well) from each group were plated into a 96-well plate. A total of 20 μl MTS reagent was added to each well containing 100 μl culture media. The plate was incubated for 2 h at 37°C in a humidified atmosphere containing 5% CO_2_. The optical density of the samples was then measured at a wavelength of 490 nm using a plate reader (Molecular Devices, Sunnyvale, CA, USA).

### Spheroid formation assay

The self-renewing capabilities of the cells were assessed using ultra-low attachment surface 96-well culture dishes (Corning, Inc., Corning, NY, USA). The cells were re-suspended in 200 μl serum-free medium DMEM/F12 (Gibco Life Technologies) supplemented with 20 ng/ml human epidermal growth factor (Peprotech, Inc., Rocky Hill, NJ, USA), 20 ng/ml human basic fibroblast growth factor (Peprotech, Inc.) and 1% N-2 supplement (Gibco Life Technologies), at a density of 200 cells in each well. Phase-contrast images were captured seven days later using an Olympus fluorescence microscope (Olympus, Tokyo, Japan).

### Scratch wound healing assay

Uniform wounds were scraped across plated MG63 cells, which had been grown on plastic six-well plates, using a pipette tip, prior to transfection. The initial gap length (0 h) and the residual gap length (48 h) after wounding were calculated from photomicrographs using an Olympus fluorescence microscope (Olympus).

### Matrigel™ invasion assay

A cell invasion assay was carried out using a modified Boyden Chamber consisting of Transwell^®^-precoated Matrigel™ membrane filter inserts, with 8 mm pores in 24-well tissue culture plates (BD Biosciences). Minimum Essential Medium supplemented with 10% fetal bovine serum was added to the lower chamber, and served as the chemoattractant.

### Statistical analysis

Differences between the groups were analyzed using Student’s t-test. Correlations between gene expression levels were studied using Pearson’s correlation. Statistical analyses were performed using SPSS version 18.0 (SPSS, Inc., Chicago, IL, USA). For all statistical analyses, P<0.05 was considered to indicate a statistically significant difference.

## Results

### Expression of HIF2PUT is correlated with HIF-2α in osteosarcoma

**Figure 1:**
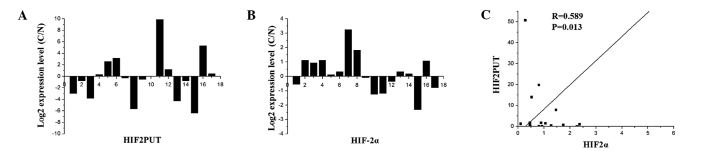
Correlation between long non-coding (lnc)RNA-hypoxia inducible factor (HIF)-2α promoter upstream transcripts (HIF2PUT) and mRNA HIF-2α expression levels in osteosarcoma tissue. (A) lncRNA-HIF2PUT and (B) mRNA HIF-2α expression levels were analyzed by quantitative polymerase chain reaction in 17 osteosarcoma tissue samples. (C) Expression levels of lnc-HIF2PUT were positively correlated with HIF-2α mRNA expression levels (P<0.05).

HIF2PUT and HIF-2α expression levels were assessed in a group of 17 patients with osteosarcoma. For each patient, RNA was isolated from cancerous and adjacent non-tumorous osteosarcoma tissues. The expression levels of HIF2PUT were significantly correlated with those of HIF-2α in the osteosarcoma tissue samples (R=0.589, P<0.05, Fig. 1A–C).

**Figure 2:**
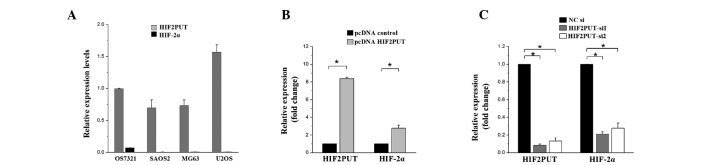
Overexpression or knockdown of long non-coding (lnc)RNA-hypoxia inducible factor (HIF)-2α promoter upstream transcripts (HIF2PUT) co-regulates mRNA expression levels of HIF-2α. (A) lncRNA-HIF2PUT and mRNA HIF-2α expression levels were analyzed by quantitative polymerase chain reaction in four osteosarcoma cell lines. (B) Expression levels of HIF2PUT and HIF-2α were significantly elevated in the pcDNA-HIF2PUT group, compared with the negative control in transfected MG63 cells (^*^P<0.05). (C) Expression levels of HIF2PUT and HIF-2α were significantly reduced in the HIF2PUT small interfering (si)RNA groups, as compared with the negative control (NC) group in transfected MG63 cells (^*^P<0.05).

HIF-2α mRNA expression was co-regulated with the lncRNA HIF2PUT overexpression or knockdown in the MG63 cells. HIF2PUT and HIF-2α expression levels were analyzed by qPCR in four osteosarcoma cell lines. The expression levels of HIF-2α were low in all four cell lines (Fig. 2A), whereas the expression levels of HIF2PUT were notably higher. The MG63 cells were chosen as the candidate cell line for the subsequent HIF2PUT overexpression and knockdown experiments, since the expression levels of HIF2PUT in this cell line were relatively moderate compared with the other cell lines. In the overexpression experiment, a HIF2PUT cDNA plasmid was constructed and stably transfected into the MG63 cells. lncRNA HIF2PUT expression levels were markedly upregulated, and HIF-2α mRNA expression levels were also moderately elevated (Fig. 2B). In the knockdown experiment, transfection of the cells with HIF2PUT siRNA resulted in a significant downregulation of HIF2PUT expression levels, and HIF-2α mRNA expression levels were also markedly decreased (Fig. 2C).

### Transfection with HIF2PUT siRNA enhances the proliferation, migration and self-renewal of MG63 cells

**Figure 3:**
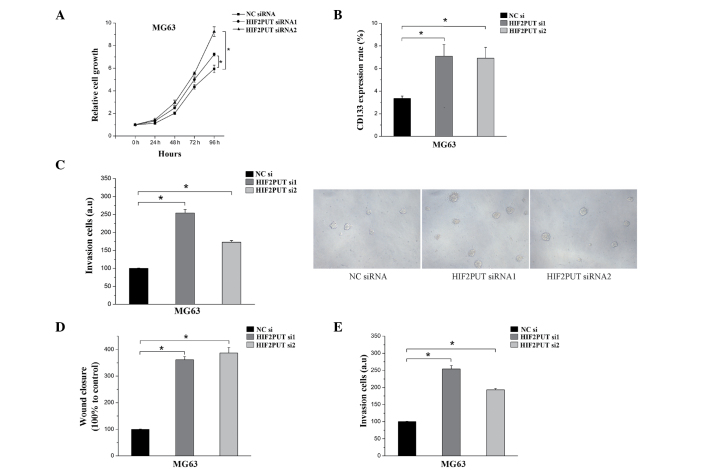
Knockdown of long non-coding (lnc)RNA-hypoxia inducible factor-2α promoter upstream transcripts (HIF2PUT) by small interfering (si)RNA enhanced the proliferation, migration and self-renewal of MG63 osteosarcoma cells. (A) Cell growth was elevated by HIF2PUT siRNA in MG63 cells. HIF2PUT siRNA groups exhibited elevated growth compared with the negative control (NC) group (^*^P<0.05). (B) Knockdown of HIF2PUT enhanced the percentage of CD133^+^ cancer stem cells (CSCs) in the MG63 cells, as determined by flow cytometry (^*^P<0.05). (C) Knockdown of HIF2PUT enhanced the self-renewal of CSCs. Spheroid-formation assay showed that the self-renewal capacities of the HIF2PUT siRNA groups were markedly enhanced (^*^P<0.05). (D) Scratch wound healing and (E) invasion assays demonstrated that the migratory capacities of the HIF2PUT siRNA groups were markedly increased (^*^P<0.05).

The proliferation of MG63 cells was evidently accelerated following knockdown of HIF2PUT expression (Fig. 3A), as determined by MTS assay. The FCM analysis demonstrated that the expression rate of the stem cell marker CD133 was significantly elevated in the MG63 cells following transfection with HIF2PUT siRNA (Fig. 3B). These results coincided with an increased rate of spheroid-formation in the stem cell culturing media (Fig. 3C). To further investigate the effects of HIF2PUT on cell migration and invasion, scratch wound healing and Matrigel™ invasion assays were performed on the MG63 cells *in vitro*. The wound healing and Matrigel™ invasion assays demonstrated a marked elevation of cell migration in the cells transfected with the HIF2PUT siRNAs, as compared with the negative control group (Fig. 3D and E).

### Overexpression of HIF2PUT decreases the proliferation, migration and self-renewal of MG63 cells

**Figure 4:**
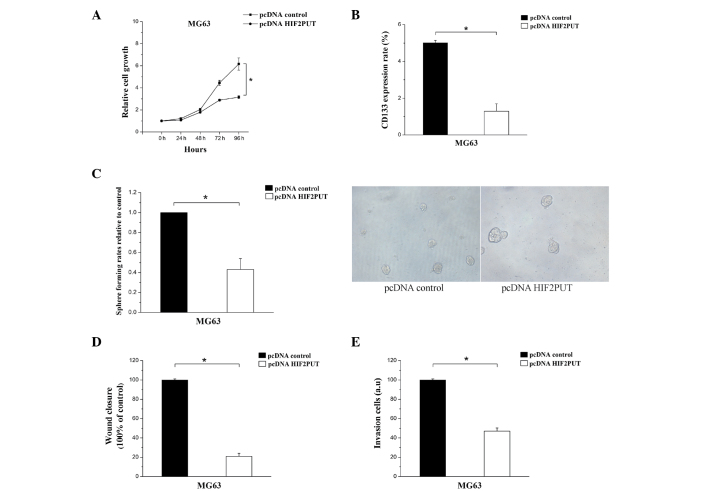
Overexpression of long non-coding (lnc)RNA-hypoxia inducible factor (HIF)-2α promoter upstream transcripts (HIF2PUT) decreased the proliferation, migration and self-renewal of MG63 osteosarcoma cells.(A) Cell growth was inhibited by transfection of MGG3 osteosarcoma cells with pcDNA-HIF2PUT, as compared with the pcDNA control group (^*^P<0.05). (B) Overexpression of HIF2PUT decreased the percentage of CD133^+^ cancer stem cells (CSCs), as determined by flow cytometry (^*^P<0.05). (C) Overexpression of HIF2PUT inhibited the self-renewal of CSCs. Spheroid formation assay showed that the self-renewing capacity of the pcDNA-HIF2PUT group was markedly inhibited (^*^P<0.05). (D) Scratch wound healing and (E) invasion assays showed that the migratory capacity of the pcDNA-HIF2PUT group was markedly decreased (^*^P<0.05).

To further identify the role of HIF2PUT in MG63 cells, functional assays were performed with MG63 cells with an overexpression of HIF2PUT (pcDNA-HIF2PUT), as compared with the MG63 cells transfected with pcDNA-control. The MG63 cells transfected with pcDNA-HIF2PUT had a significantly slower rate of growth, as compared with the cells transfected with the pcDNA-control (Fig. 4A). To determine the effects of HIF2PUT overexpression on CSCs in MG63 cells *in vitro*, the percentage of CD133^+^ expressing cells was determined by FCM, and the stem cell sphere-forming ability was assessed by spheroid-formation assay. Following transfection with pcDNA-HIF2PUT, the percentage of CD133^+^ CSCs and the number of stem cell spheres were significantly decreased in MG63 cells *in vitro* (Fig. 4B and C). Furthermore, the migratory and invasive ability of the cells was markedly inhibited by pcDNA-HIF2PUT transfection (Fig. 4D and E).

## Discussion

Osteosarcoma, and other types of cancer, contains a small subset of cells with stem cell-like properties, which are responsible for tumor aggression and recurrence (20). An improved understanding regarding the molecular network modulating these stem cell characteristics is essential for the development of effective therapeutic strategies for osteosarcoma.

Recently, growing evidence has indicated that the human genome is largely transcribed into noncoding RNAs, such as microRNAs and lncRNAs. lncRNAs may have critical regulating roles in the extensive biological processes of certain cells, including stem cells (10). Previous studies have suggested that lncRNAs may act as crucial modulators in cancer development (7–9).

However, the functional role of lncRNAs in CSCs remains unknown, particularly in osteosarcoma. In the present study, by loss- and gain-of function experiments, a novel lncRNA, HIF2PUT, was shown to inhibit the proliferation, self-renewal and migration of osteosarcoma stem cells. CD133 is considered a CSC marker of osteosarcoma (20). The present study showed that overexpression of HIF2PUT markedly decreased the CD133^+^ cell population in MG63 cells. These results suggest that HIF2PUT may negatively modulate the CSC population in osteosarcoma. The present study may provide the first CSC-associated lncRNA: HIF2PUT, in osteosarcoma, which may be a potential therapeutic target.

HIF2PUT is a PROMPT of HIF-2α. The cellular biological effects of PROMPTs in the human body have yet to be elucidated (8). Previous studies have suggested that PROMPTs can bind other proteins. Growth arrest-specific 5 is an important cancer associated lncRNA that can regulate downstream genes by binding to the transcription factor, ensuring the transcription factor can no longer bind to promoters (22). However, other studies have suggested that the majority of PROMPTs exert their function by regulating the transcriptional activity of their host PCGs (11–14).

The present study investigated the association between HIF2PUT and its host PCG, HIF-2α. The expression levels of HIF2PUT were shown to be positively correlated with those of HIF-2α in osteosarcoma tissues. Furthermore, in the MG63 cells, downregulation of HIF2PUT expression resulted in a concomitant decrease in the mRNA expression levels of HIF-2α. These results suggest that the HIF2PUT may be a regulator of HIF-2α in osteosarcoma.

It has previously been reported that transcripts of HIF-2α are restricted to particular cell types (15). The present study showed that expression of HIF-2α in osteosarcoma tissues can be detected; however, the expression levels in all four osteosarcoma cell lines were low. This may be due to the osteosarcoma tissues containing not only tumor cells, but also numerous types of supporting cells. Knowles *et al* (23), detected low expression levels of HIF-2α in MG63 cells in non-hypoxic conditions; however, the expression levels were higher in hypoxic conditions, which coincides with the results of the present study.

Previous studies have indicated that the role of HIF-2α may vary from cancer gene to tumor suppressor in numerous types of cancer (17,24–26). The present study could not identify the exact function of HIF-2α in osteosarcoma, due to the extremely low expression. In conclusion, the results of the present study may provide primary evidence that lncRNA-HIF2PUT can suppress the properties of CSCs in osteosarcoma, partly by regulating the expression of HIF-2α. Further work targeting this novel lncRNA in osteosarcoma may provide potential therapeutic targets in the future.

## Figures and Tables

**Table I:** Characteristics of the patients participating in the present study.

Number	Age	Sex	Histological diagnosis	Stage	Tumour location
1	25	F	osteosarcoma	II A	Distal end of femur
2	20	M	osteosarcoma	II B	Proximal end of tibia
3	14	M	osteosarcoma	II B	Proximal end of humerus
4	28	M	osteosarcoma	II B	Distal end of tibia
5	8	M	osteosarcoma	I B	Distal end of femur
6	15	M	osteosarcoma	II A	Proximal end of tibia
7	14	F	osteosarcoma	I B	Distal end of femur
8	10	M	osteosarcoma	I A	Distal end of femur
9	15	M	osteosarcoma	II A	Proximal end of tibia
10	15	F	osteosarcoma	I B	Distal end of femur
11	11	M	osteosarcoma	II A	Distal end of femur
12	11	M	osteosarcoma	II A	Proximal end of tibia
13	12	F	osteosarcoma	II B	Proximal end of tibia
14	18	M	osteosarcoma	II A	Distal end of femur
15	12	M	osteosarcoma	II B	Proximal end of tibia
16	21	F	osteosarcoma	I B	Distal end of femur
17	19	M	osteosarcoma	II A	Proximal end of tibia

Staging is based on Enneking system staging. F, female; M, male.

**Table II:** Primers for quantitative polymerase chain reaction analysis.

Gene	Forward	Reverse
β-actin	5′-CCACTGGCATCGTGATGGA-3′	5′-CGCTCGGTGAGGATCTTCAT-3′
HIF2PUT	5′-CGGAGGTGTTCTATGAGCTGG-3′	5′-AGCTTGTGTGTTCGCAGGAA-3′
HIF-2α	5′-TGGGATCTAACAGGAACAGC-3′	5′-CTAAATAGCCAGACAAGGGT-3′

HIF2PUT, HIF-2α promoter upstream transcript; HIF-2α, hypoxia-inducible factor-2α.
